# Recombinant human erythropoietin *α* modulates the effects of radiotherapy on colorectal cancer microvessels

**DOI:** 10.1038/sj.bjc.6603568

**Published:** 2007-02-13

**Authors:** W Ceelen, T Boterberg, P Smeets, N Van Damme, P Demetter, O Zwaenepoel, L Cesteleyn, P Houtmeyers, M Peeters, P Pattyn

**Affiliations:** 1 Department of Surgery, Ghent University Hospital, Ghent, Belgium; 2 Department of Radiotherapy, Ghent University Hospital, Ghent, Belgium; 3 Department of Radiology, Ghent University Hospital, Ghent, Belgium; 4 Department of Gastroenterology, Ghent University Hospital, Ghent, Belgium; 5 Department of Pathology, Erasmus University Hospital, Université Libre de Bruxelles, Brussels, Belgium

**Keywords:** erythropoietin, radiothkerapy, oxygenation, colorectal, microcirculation

## Abstract

Recent data suggest that recombinant human erythropoietin (rhEPO) modulates tumour growth and therapy response. The purpose of the present study was to examine the modulation of radiotherapy (RT) effects on tumour microvessels by rhEPO in a rat colorectal cancer model. Before and after 5 × 5 Gy of RT, dynamic contrast-enhanced -magnetic resonance imaging was performed and endothelial permeability surface product (PS), plasma flow (*F*), and blood volume (*V*) were modelled. Imaging was combined with pO_2_ measurements, analysis of microvessel density, microvessel diameter, microvessel fractal dimension, and expression of vascular endothelial growth factor (VEGF), hypoxia-inducible factor-1 *α* (HIF-1*α*), Bax, and Bcl-2. We found that RT significantly reduced PS and *V* in control rats, but not in rhEPO-treated rats, whereas *F* was unaffected by RT. Oxygenation was significantly better in rhEPO-treated animals, and RT induced a heterogeneous reoxygenation in both groups. Microvessel diameter was significantly larger in rhEPO animals, whereas VEGF expression was significantly lower in the rhEPO group. No differences were observed in HIF-1*α*, Bax, or Bcl-2 expression. We conclude that rhEPO results in spatially heterogeneous modulation of RT effects on tumour microvessels. Direct effects of rhEPO on neoplastic endothelium are likely to explain these findings in addition to indirect effects induced by increased oxygenation.

Anaemia commonly occurs in colorectal cancer patients especially if they are treated with neoadjuvant radiotherapy (RT) or chemotherapy. Anaemia not only adversely affects the clinical condition of these patients, but also contributes to the development of tumour hypoxia, recognised as a major negative determinant of sensitivity to RT, chemoradiotherapy, and certain chemotherapeutic agents (Harrison and Blackwell, 2004; van Halteren *et al*, 2004).

Recent clinical studies have shown that administration of recombinant human erythropoietin (rhEPO, epoetin alfa) increases haemoglobin levels and improves quality of life in patients with cancer-related anaemia (Littlewood *et al*, 2001). Over the last decade, it has become clear that the action or rhEPO extends into a wide range of cellular mechanisms involved in stem cell development, maintenance of cellular integrity, and physiological angiogenesis (Maiese *et al*, 2005). The demonstration of the erythropoietin (EPO) receptor in various neoplastic tissues and the observation in a recent clinical trial that mortality was higher in nonanaemic rhEPO-treated breast cancer patients highlighted the possible effects of rhEPO on tumour growth and angiogenesis (Leyland-Jones, 2003). Preclinical studies investigating the role of EPO and EPO–EPO receptor signalling on tumour growth and angiogenesis have yielded contradictory results. Yasuda *et al* (2003) noted the inhibition of angiogenesis and tumour cell survival in stomach and melanoma xenografts following blockade of EPO signalling. The results of Hardee *et al* (2005), however, suggested that administration of rhEPO did not affect angiogenesis or tumour growth in human colon and head and neck xenografts.

The importance of tumour oxygenation for RT response is well established, and there has been considerable interest in modulating tumour oxygenation and RT response by rhEPO administration. Experimentally, exogenous rhEPO has been shown to improve or restore radioresponsiveness in both anaemic and nonanaemic animals (Thews *et al*, 1998; Stuben *et al*, 2003; Pinel *et al*, 2004). Interestingly, darbepoetin alfa, an EPO analogue with a longer half-life, did not enhance radioresponsiveness in a rat mammary adenocarcinoma model (Kirkpatrick *et al*, 2006).

The exact mechanism by which rhEPO exerts its effects on tumour oxygenation is at present unclear. Indeed, recent data suggest that this effect may be independent of changes in haemoglobin and mediated by changes in vascular endothelial growth factor (VEGF) expression and microvessel morphology (Blackwell *et al*, 2003; Tovari *et al*, 2005).

We aimed to further characterise the effects of rhEPO on microvascular morphology and function in non-anaemic rats using a novel imaging methodology. We previously used dynamic contrast-enhanced magnetic resonance imaging (DCE-MRI) with a macromolecular contrast agent (CA) to demonstrate a significantly decreased neovascular leakage after fractionated RT in a rat colorectal cancer model (Ceelen *et al*, 2006). Dynamic contrast-enhanced magnetic resonance imaging allows noninvasive *in vivo* study of microvascular properties of a complete tumour, thereby taking into account the important spatial heterogeneity of solid tumours with zones of well perfused tissue, as well as hypoxic or necrotic areas (Hylton, 2006). Here, we studied the effects of rhEPO on RT-induced microenvironmental changes in a rat colorectal cancer model and correlated noninvasively obtained data with invasive oxygenation and flow measurements, microvessel density, complexity and diameter, and expression of hypoxia-regulated and apoptosis markers.

## MATERIALS AND METHODS

The experimental protocol was approved by the Animal Experimentation Ethical Committee of the Ghent University, Ghent, Belgium.

### Animal and tumour model

Male Wag/Rij rats were bought from Harlan (Horst, The Netherlands). The CC531 cell line is a 1,2-dimethylhydrazine-induced, moderately differentiated, and weakly immunogenic colon adenocarcinoma, syngeneic with WAG/Rij rats. This cell line is well studied and has been proven to provide a tumour–host model similar to human colorectal carcinogenesis (Hagenaars *et al*, 2000). Cells were grown in plastic culture flasks in RPMI 1640 medium, buffered with HEPES (20 mM) (Invitrogen Corporation, Gibco, Ghent, Belgium) additionally supplemented with 10% fetal calf serum, 4 mM L-glutamine, 50 U ml^−1^ penicillin, and 50 *μ*g ml^−1^ streptomycin at 37°C in a humidified atmosphere with 5% CO_2_ in air. The cells were transferred at 95% confluency. Two million cells suspended in 0.2 ml of saline were injected subcutaneously (s.c.) in the proximal hind leg. Tumours reached a size of 0.5–1 cm after a period of 4 weeks. Once a tumour growth of minimally 8 mm diameter was observed, a jugular vein catheter was inserted and tunnelled to the interscapular region. To maintain catheter patency, continuous infusion at 0.5 ml saline per hour was administered with a cage-mounted swivel and a flexible metal tether system (Uno BV, Didam, The Netherlands) allowing the animal full mobility.

### Experimental therapy

Recombinant human EPO has been shown to bind to the rodent EPO receptor (Okano *et al*, 1993). Animals were randomly divided into two groups: a control group (*n*=11) and an rhEPO group (*n*=15) receiving rhEPO (Eprex®, Janssen Cilag, Beerse, Belgium) at a dose of 3 × 0.1 ml (286 IU) s.c. per week. The dosage was based on a dose-finding study during which five or eight rhEPO administrations weekly resulted in an excessive haematocrit rise and important mortality (data not shown).

Rats were longitudinally studied during 3 weeks using the following timeframe: start of rhEPO administration (day 1); first DCE-MRI, oxygenation and flow measurement (day 8); fractionated RT 5 × 5 Gy (day 13–17); second DCE-MRI, oxygenation and flow measurement, and killing by anaesthesia overdose and excision of tumours for histology (all on day 22).

### Radiotherapy

Rats were not sedated and the tumour-bearing hind leg was immobilised using a Plexiglass holder, as described previously (Ceelen *et al*, 2001; El-Malt *et al*, 2001). Briefly, rats were placed in a purpose-built Plexiglass holder in prone position. The hind legs were pulled through an opening in the holder and immobilised. Before each fraction, a radiation field was simulated encompassing the tumour with a margin of 1.5 cm. Photon irradiation was performed with a 5 MV linear accelerator (Elekta, Crawley, UK). Five fractions of 5 Gy (total dose 25 Gy) were delivered on five consecutive days. As the tumours were inoculated s.c., they were covered with tissue-equivalent silicone bolus of 1 cm to prevent the build-up effect under the skin. One single direct field at a fixed source-skin distance of 100 cm was used. The dose was calculated to the midpoint of the tumours according to their volume in each individual animal, as obtained during simulation. Dynamic contrast-enhanced magnetic resonance imaging and oxygenation measurements were performed 5 days before and 5 days after the completion of RT.

### Magnetic resonance imaging

The principle of DCE-MRI consists of serial measurements of signal intensity changes in both tumour tissue and a feeding artery after bolus injection of a paramagnetic CA. Depending on the physical properties of the CA and the leakiness of the microvessel wall, a fraction of the CA will reach the interstitial space of the tumour, where an increase in signal intensity over time will be observed. After translation of signal intensity changes to CA concentration values, pharmacokinetic modelling allows calculation of physiological properties such as microvessel permeability and tumour blood volume.

Dynamic contrast studies were performed with P792, a new monogadolinated rapid clearance MRI blood-pool CA, which is cleared by renal elimination. The molecular weight of the compound is 6.47 kDa, but the mean diameter of P792 is 50.5 Å and the T1 relaxivity of this agent is 29 mM^−1^ s^−1^ at 60 MHz (Port *et al*, 2001a). The apparent hydrodynamic volume of P792 is 125 times greater than that of Gd-DOTA (gadoterate meglumine, Dotarem) and as a result of this high molecular volume, P792 is characterised by a limited diffusion across the normal endothelium and therefore ideally suited to study hyperpermeable neoplastic vessels (Port *et al*, 2001b). Experimentally, P792 has been used to study permeability effects of anti-angiogenesis therapy in a prostate cancer model (Pradel *et al*, 2003). We have previously demonstrated that P792 selectively enhances tumour tissue in this colorectal cancer model (Ceelen *et al*, 2006).

T_1_-weighted DCE-MRI was performed on a Siemens Magnetom Symphony® 1.5 T scanner (Siemens AG, Erlangen, Germany). Animals were sedated with 0.2–0.4 ml of medetomidine (Domitor®, Novartis Animal Health, Basel, Switzerland). Imaging comprised a single axial slice that was positioned through both lower limbs and the centre of the tumour. Before the contrast series, *T*_1_ zero time maps were constructed from two spin echo sequences with different repetition times (TR 1000 and 318 ms, respectively). Details of this sequence were as follows: slice thickness 3 mm, field of view (FOV) 140 × 88, matrix size 256 × 160, echo time (TE) 20 ms, and flip angle 90°. Dynamic imaging was performed with a 4-antenna wrist coil (diameter 10 cm) using an inversion recovery TurboFLASH sequence. Details of the pulse sequence were as follows: temporal resolution 1.1 s (i.e. TR 1100 ms), FOV 140 × 88, matrix size 256 × 160, slice thickness 5 mm, TE 4.08 ms, inversion time 560 ms, and flip angle 12°. A bolus of 0.3–0.4 ml of P792 was manually injected as fast as possible (approximately 1 ml s^−1^) through a central venous line after the fourth scan. A total of 500 images was obtained for a total scan time of 550 s.

### Tracer kinetic modelling

**Figure 1:**
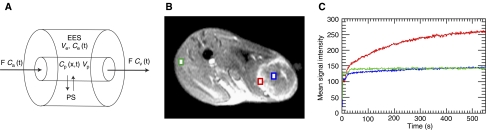
Illustration of the St Lawrence and Lee tissue homogeneity model applied to dynamic MRI data. (**A**) Two-compartment model used for kinetic modelling. Following injection, CA enters the tissue capillary unit (length×) and distributes between the vascular space and the EES through a semipermeable vessel wall characterised by a permeability surface product PS. Measurement of the temporal changes in CA concentration (calculated from changes in MRI signal intensity) in both the vascular space and the EES allows to calculate the *F*, *V*_p_, and PS. (**B**, **C**) Examples of contrast enhancement curves (500 images with 1.1 s temporal resolution) observed in regions of interest selected in the tumour rim (red), tumour core (blue), and normal muscle. Normal microvessels do not leak the macromolecular contrast agent. The colorectal cancer is grown subcutaneously in the left lower limb.

Pixel-by-pixel pharmacokinetic modelling of DCE-MRI data was performed with the research mode of a dedicated software package (MIStar®, Apollo Medical Imaging, Melbourne, Australia). Extraction of both microvascular permeability and flow data was based on the tissue homogeneity (TH) model of capillary exchange originally described by Johnson and Wilson (1966) and later adapted for the study of cerebral flow by St Lawrence and Lee (1998); . This model (Figure 1) consists of a plasma space, in which the CA concentration is a function of both time and distance along the capillary unit, and an extracellular extravascular space (EES) assumed to be homogenously mixed (i.e. a compartment). Leakage of CA takes place between the vascular space and EES through a semipermeable membrane characterised by a permeability surface product (PS). As P792 does not enter the intracellular compartment, the sum of the fractional plasma volume (*V*_p_) and fractional extracellular volume (*V*_e_) reached by the CA equals 100%, that is, *V*_p_+*V*_e_=1.

When the assumption is made that changes in EES concentration per unit time are negligible compared with changes in plasma concentration, an adiabatic approximation to the TH model can be derived, which has been used in modelling of DCE-MRI CA kinetics applied to tumour microvasculature (St Lawrence and Lee, 1998; Henderson *et al*, 2000).

The adiabatic approximation to tissue homogeneity (AATH) model is given by superposition of the arterial input function (AIF) with a time-varying residue function: 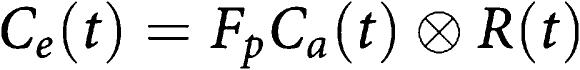 where *C*_e_ (mM) is the CA concentration in the EES, *F*_p_ (unitless) is the plasma flow, *R* denotes the residue function, and ⊗ is the convolution operator.

The time course of CA arrival is divided in a vascular phase (*t*<*τ*, with tau as the transit time through a capillary) and a tissue phase (*t*>*τ*). Depending on the time interval, the residue function will encompass a vascular and a tissue component: 
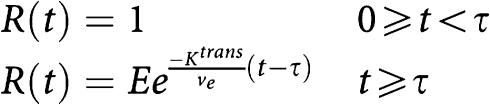
 with *E* the extraction ratio from plasma space to EES, *K*^trans^ the endothelial transfer constant (min^−1^), *v*_e_ the fraction of the EES available as leakage space (unitless), and *τ* is the mean capillary transit time (s).

**Figure 2:**
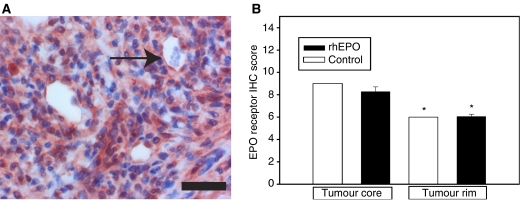
Immunoreactivity of the erythropoietin receptor. (**A**) Representative immunohistochemistry showing both cytoplasmatic and membrane staining of tumour cells and endothelial cells (arrow). Bar, 25 *μ*m. (**B**) Comparison of EPO-R immunoreactivity scores in the tumour rim and core of both control and rhEPO-treated animals. Columns, mean; bars, s.e.; ^*^P<0.001 *vs* the tumour core.

The tissue CA concentration can therefore be modelled as 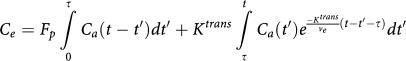 In each animal, the pixel containing the AIF curve was selected in the femoral artery feeding the tumour-bearing limb. A region of interest (ROI) was drawn encompassing the outer vascular rim of each tumour. Within this ROI, a pixel by pixel curve-fitting routine based on the Levenberg–Marquardt minimisation method was performed, generating parametric maps (Figure 2) of the following parameters: *F*_p_, PS, and the fractional plasma volume *V*_p_, calculated as *F*_p_ × MTT. Numerical parameter values for each pixel were exported to a spreadsheet for statistical analysis.

### Tissue pO_2_ and flow measurements

Tissue oxygenation and laser Doppler flow (LDF) were measured with a fibreoptic probe combining fluorescence quenching with laser Doppler flowmetry (OxyLite® and OxyFlo®, Oxford Optronix, Oxford, UK) (Blackwell *et al*, 2003; Brurberg *et al*, 2004). A precalibrated fibreoptic probe was inserted 5 mm deep into the tumour using a Seldinger technique; the probe was then withdrawn in 40 steps of 100 *μ*m each over a total distance of 4 mm using a micromanipulator (model MN151, Narishige International Ltd, London, UK). After each micromanipulator movement, measurements were started as soon as a stable reading was obtained. Tissue pO_2_ was sampled every 2 s. Over this 4 mm trajectory, oxygenation and LDF values were recorded separately for the tumour core (central 1–2 mm) and peripheral angiogenic rim (outer 1 mm). Tissue pO_2_ was expressed in mmHg, whereas LDF was expressed in arbitrary units (a.u.).

### Immunohistochemistry

Paraffin-embedded tissue samples were used for immunohistochemistry with the following antibodies: anti-EPO receptor (M-20) (sc-697, Santa Cruz Biotechnology Inc., Santa Cruz, CA, USA), anti-Bcl-2 (sc-7832, Santa Cruz), anti-Bax (sc-7480, Santa Cruz), anti-VEGF (sc-7269, Santa Cruz), and anti-hypoxia-inducible factor-1 *α* (HIF-1*α*) (sc-10790, Santa Cruz). Paraffin-embedded sections were rehydrated by serial immersion in xylene and ethanol. After rinsing, the endogenous peroxidase was blocked with 0.3% hydrogen peroxide. The sections were subsequently incubated with a biotinylated secondary antibody, followed by incubation with a streptavidin–peroxidase complex (LSAB+kit, Dako, Heverlee, Belgium). The colour reaction was developed using 3-amino-9-ethylcarbazole substrate (Dako) as chromogen. Finally, the sections were counterstained with haematoxylin. Semiquantitative scoring was based on a method modified after Coppola *et al* (1998), with a scale ranging from 0 to 9. The scale was based on scoring of the fraction of positive cells (0: all cells negative; 1: <33% positive; 2: 33–66% positive; 3: >66% positive) and the staining intensity (1: weak; 2: moderate; 3: intense). Both scores were multiplied to a maximum score of 9. Scoring was performed separately on the tumour core and peripheral tumour rim.

### Microvascular density and diameter

Microvascular density (MVD) was determined with a method modified after Weidner *et al* (1991). After incubating 5 *μ*m frozen slices with anti-CD31 antibodies (TLD-3A12, Serotec, Oxford, UK), the entire tumour section was scanned at low-power (objective, × 4) to identify ‘hot spots’, which are the areas of highest neovascularisation. Individual microvessels were then counted under higher power (objective, × 40) to obtain a vessel count in a defined area, and the average vessel count in three hot spots was taken as the MVD.

Microvascular diameter was measured on digitised CD31-stained slices (objective, × 10). From each rat, five different zones were analysed and the largest diameter was measured from all visible microvessels using NIH ImageJ software (version 1.35p, available from http://rsb.info.nih.gov/ij).

### Microvessel fractal dimension

The tumour-associated microvascular network can be considered as a complex architecture defined not only by the number of microvessels but also by the degree of branching, tortuosity, and irregularity. Fractal analysis of two-dimensional histology slides has been shown to provide additional information on tumour microvascular complexity (Sabo *et al*, 2001; Dey, 2005). Whereas classical geometrical objects are usually associated with integer values for a dimension (1 for a line, 2 for a square), complex biological structures are best defined by a fractal dimension that is a rational number between 1 and 2. The more complex (branched, tortuous) the microvascular structure, the closer the fractal dimension is to 2.

From each tumour, digital images were obtained from five CD31-stained tumour hot spots (objective, × 10) and analysed with ImageJ software. By applying a colour threshold, non-CD31-stained pixels were removed from the image. The microvessel fractal dimension (MFD) was calculated using the box-counting method. The image is divided into increasingly smaller boxes, and after each step the number of nonempty boxes is counted.

The fractal dimension is calculated as 
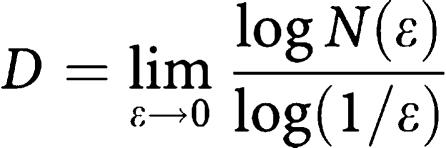
 with *ε* the length of a box side and *N*(*ε*) the smallest number of boxes required to contain all CD31-stained pixels. In practice, the limit of a box sized 0 cannot be applied and therefore the MFD was calculated as the slope of the curve fitted after plotting log *N*(*ε*) *vs* log (1/*ε*). Calculations were performed with the fractal dimension plugin available in the ImageJ environment.

### Statistical analysis

Data are expressed as mean±standard error (s.e.) of the mean, unless stated otherwise. Differences between two groups of continuous data were analysed with the Student's *t*-test or, when data distribution was non-Gaussian, with the nonparametric Mann–Whitney *U*-test, whereas differences between fractions were evaluated with the *χ*^2^ or Fisher's exact test. Statistical significance was assumed when *P*⩽0.05. All calculations and plotting were performed with SigmaStat software (version 3.11, Systat Software, Richmond, VA, USA).

## RESULTS

### Expression of the EPO receptor

All tumours showed expression of the EPO-R on neoplastic cells and neoplastic endothelium (Figure 2). There was no difference in expression score between the control and rhEPO-treated animals. In both groups, however, EPO-R immunoreactivity was significantly higher in the tumour core compared to the vascular tumour rim.

### Effects of rhEPO on haematocrit

Haematocrit values were tested before starting the experiment and on day 8 (immediately before the first MR imaging and oxygenation measurements). In rhEPO-treated rats, mean haematocrit showed a 25% increase from 50.7±0.4% before therapy to 62.6±0.4% on day 8 (*P*<0.001, Student's *t*-test). In the control group, haematocrit values remained unchanged.

### Effects of rhEPO on tumour growth

The present experiment was intended to detect early microvascular changes after fractionated RT and not to study the effects of rhEPO on tumour growth or modulation of RT effects on tumour growth. Administration of rhEPO was therefore started after a tumour had developed. Tumour volume before RT was 1±0.2 cm^3^ in the control group and 0.97±0.11 cm^3^ in the rhEPO group (*P*=0.79, Mann–Whitney *U*-test). After RT, tumour volume was 0.99±0.2 cm^3^ in the control group and 0.99±0.16 cm^3^ in the rhEPO group (*P*=0.99, Mann–Whitney *U*-test).

### Effects of rhEPO on RT-induced tumour microvascular changes

Pixel-by-pixel kinetic modelling encompassing both the tumour core and rim was performed with estimation of three microvascular parameters: plasma flow *F*_p_, permeability surface product PS, and fractional plasma volume *V*_p_.

**Figure 3:**
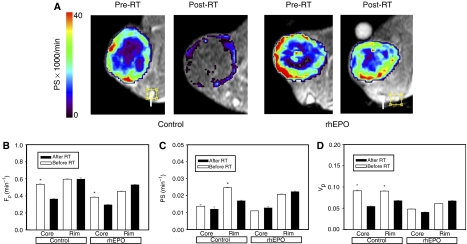
Results of kinetic modelling of DCE-MRI data. (**A**) Example of parametric maps of the permeability surface product (PS) in control animals and rhEPO-treated animals before and after RT. The tumour is masked and the arrow points to the pixel containing the selected vascular input function. (**B**–**D**) Combined kinetic data for *F*_p_, PS, and *V*_p_. Columns, mean; bars, s.e.; ^*^P<0.001.

Results of DCE-MRI microvascular data modelling are shown in Figure 3. Microvascular plasma flow in the tumour core of control rats was significantly lower after RT (*P*<0.001, Student's *t*-test). No significant effect of RT was noted in the tumour rim of control animals. In rhEPO-treated rats, microvascular flow in the tumour core was also significantly lower after RT (*P*<0.001, Student's *t*-test) but unaffected in the tumour rim. The magnitude of the RT effect on tumour core flow did not differ significantly between control and rhEPO-treated animals (33 *vs* 23% decrease, respectively, *P*=0.11, Fisher's exact test).

In control animals, the microvessel PS was significantly lowered by RT in the tumour rim (*P*<0.001, Student's *t*-test) but not in the tumour core. In rhEPO-treated animals, however, PS was not affected by RT.

Fractional plasma volume was significantly lower in both the tumour core and rim in control animals after RT (*P*<0.001, Student's *t*-test). In rhEPO-treated animals, however, no significant changes in plasma volume were noted after RT.

### Effects of rhEPO on tumour pO_2_ and LDF

**Figure 4:**
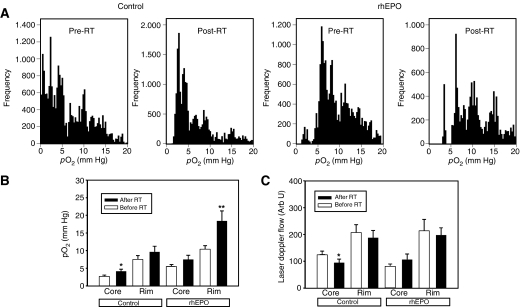
Combined results of invasive pO_2_ and laser Doppler flow measurements. Measurements were performed before and 5 days after the end of 5 × 5 Gy of RT. (**A**) Histograms of pO_2_ readings over a 4-mm trajectory in control and rhEPO-treated rats both before and after RT. A reoxygenation occurs after the end of RT, which is more pronounced in the rhEPO group. (**B**) Oxygenation data in the tumour core (central 1–2 mm) and tumour rim (superficial 1–2 mm). Columns, mean; bars, s.e.; ^*^P=0.067; ^**^P=0.032. (**C**) Laser Doppler flow data in the tumour core and rim. Columns, mean; bars, standard error; ^*^P=0.023).

Oxygenation and LDF measurements were performed before and 5 days after completion of fractionated RT. Mean pO_2_ values in the tumour core and peripheral rim before and after RT in both groups are shown in Figure 4A and B. Oxygenation was significantly better in the tumour rim compared with the tumour core of all animals (data not shown). In the control group, RT induced a reoxygenation in the tumour core (*P*=0.067, Student's *t*-test) but not in the tumour rim (*P*=0.36, Student's *t*-test). In the rhEPO group, on the contrary, no significant difference in oxygenation was observed in the tumour core (*P*=0.12, Student's *t*-test), whereas a significant increase in pO_2_ was observed in the tumour rim (*P*=0.032, Student's *t*-test). Both before and after RT, pO_2_ values were significantly higher in rhEPO-treated rats in both regions of the tumour (data not shown).

Mean LDF values (a.u.) are illustrated in Figure 4C. In all animals, LDF was significantly higher in the tumour rim compared with the tumour core. In the control group, RT significantly decreased LBF in the tumour core (*P*=0.023, Student's *t*-test), but not in the tumour rim. In the rhEPO group, however, RT did not influence LDF in either tumour zone. Before RT, LDF measured in the tumour core was significantly lower in rhEPO-treated animals (*P*=0.014, Student's *t*-test) compared with controls, whereas no significant difference was present in the tumour rim. After RT, LDF values in both tumour core and rim were not significantly different between controls and rhEPO-treated animals (data not shown).

### Effects of rhEPO on microvessel density

Mean microvascular density was 12.5±1.2 in the control group and 14.3±1.4 in the rhEPO group (*P*=0.35, Student's *t*-test). Within each group, MVD was significantly lower in the tumour core compared with the tumour rim (12.5±1.2 *vs* 7.3±0.6, *P*<0.001 in the control group and 14.3±1.4 *vs* 6.6±0.5, *P*<0.001 in the rhEPO group, Student's *t*-test).

### Effects of rhEPO on MFD and diameter

**Figure 5:**
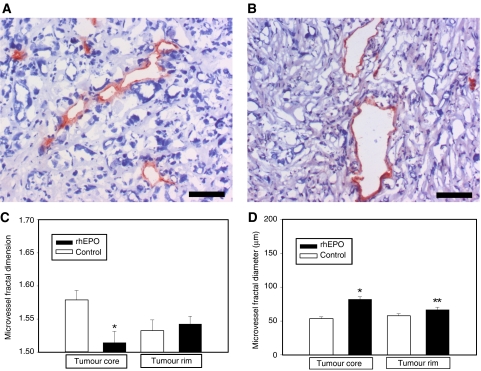
Comparison of tumour microvessel fractal dimension and diameter in control and rhEPO-treated rats. (**A**, **B**) Examples of CD31-stained microvessels in the tumour core of control and rhEPO-treated animals. Bar, 25 *μ*m. (**C**) Microvessel fractal dimension in the tumour core and rim in control and rhEPO-treated animals. Columns, mean; bars, s.e.; ^*^P=0.006. (**D**) Microvessel diameter in the tumour core and rim in control and rhEPO-treated animals. Columns, mean; bars, s.e.; ^*^P<0.001; ^**^P=0.07.

Microvessel morphology data are illustrated in Figure 5. Microvessel fractal dimension was spatially heterogeneous. In the tumour core, MFD was significantly lower in rhEPO-treated animals (*P*=0.006, Student's *t*-test). In the tumour rim, however, MFD did not differ between controls and rhEPO-treated animals (*P*=0.62, Student's *t*-test), Figure 5C.

Overall microvessel diameter (*μ*m) in tumour tissue was 55.9±2.1 in the control group and 75.4±2.7 in the rhEPO group, *P*<0.001. The increased microvessel diameter in rhEPO-treated animals was more pronounced in the tumour core (*P*<0.001, Student's *t*-test) than in the tumour rim (*P*=0.07, Student's *t*-test), Figure 5D.

### Effects of rhEPO on expression of VEGF, HIF-1*α*, Bax, and Bcl-2

**Figure 6:**
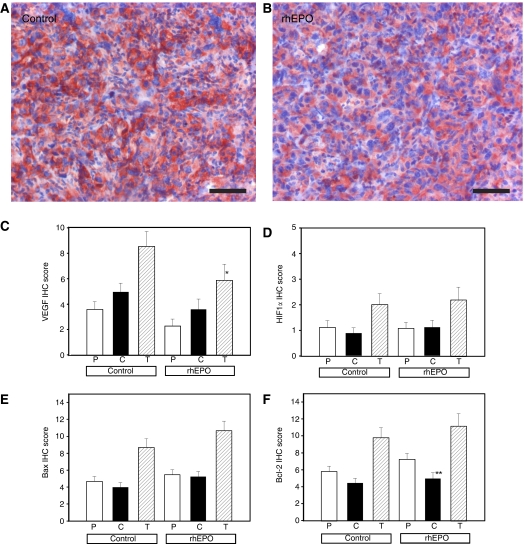
Expression of hypoxia-regulated and apoptosis-related markers. (**A**, **B**) Example of VEGF staining intensity in control and rhEPO-treated animals. Bar, 50 *μ*m. (**C**–**F**) Expression of VEGF, HIF-1*α*, Bax, and Bcl-2 in the tumour peripheral rim (P), core (**C**), and total score. Columns, mean; bars, s.e.; ^*^P=0.048; ^**^P=0.012.

Immunohistochemistry data are summarised in Figure 6. Total VEGF expression score was significantly higher in the control group (*P*=0.048, Mann–Whitney *U*-test). Within each group, the difference in VEGF expression in the tumour core *vs* tumour rim was not significant.

Total expression of HIF1*α* did not differ significantly between both groups (*P*=0.78, Mann–Whitney *U*-test). There was also no significant difference in expression of Bax (*P*=0.21, Mann–Whitney *U*-test) or Bcl-2 (*P*=0.72, Mann–Whitney *U*-test) between control and rhEPO-treated animals. In rhEPO-treated animals, Bcl-2 expression was significantly lower in the tumour core compared with the tumour rim (*P*=0.012, Mann–Whitney *U*-test).

## DISCUSSION

The presence of hypoxia adversely affects radiotherapy response and prognosis in cancer patients (Varlotto and Stevenson, 2005). As the oxygen-carrying capacity is mainly determined by the blood haemoglobin concentration, pharmacological manipulation aiming to restore or increase haemoglobin levels have received considerable interest in cancer patients undergoing RT or chemotherapy. Erythropoietin is a pleiotropic hormone whose biological role has recently been shown to extend not only to the hematopoietic tissues but also to the neuronal and cardiovascular systems, where it exerts a cytoprotective effect (Maiese *et al*, 2005). Administration of exogenous rhEPO not only improved quality of life but also increased survival in a number of clinical studies in solid tumours (Munstedt *et al*, 2004; Rades *et al*, 2006). On the other hand, EPO has been shown to exert direct effects on angiogenesis and tumour growth mediated by presence of the EPO receptor on endothelial cells and a number of malignant cell types (Jaquet *et al*, 2002; Jelkmann and Wagner, 2004). Preclinical studies investigating the effect of exogenous rhEPO on tumour growth and angiogenesis are at present inconclusive (Hardee *et al*, 2005). Similarly, whereas some preclinical data suggest that rhEPO increases response to RT, chemotherapy or photodynamic therapy, other studies did not identify any effects of rhEPO (Stuben *et al*, 2003; Pinel *et al*, 2004; Kirkpatrick *et al*, 2006). In the clinical setting, the effects of rhEPO on locoregional control and survival of cancer patients treated with RT or chemotherapy are a matter of ongoing controversy. In head and neck cancer patients undergoing RT and randomised to therapy with epoetin beta or placebo, locoregional control and survival were significantly worse in the EPO-treated group, although anemia was corrected in these patients (Henke *et al*, 2003). Similarly, the Breast Cancer Erythropoietin Trial (BEST) examining the use of rhEPO in women with metastatic breast cancer receiving first-line chemotherapy was terminated prematurely owing to an observed higher mortality in the rhEPO group (Leyland-Jones, 2003). Both trials have, however, been the subject of criticism concerning study design, conduct, and post-trial analysis.

We aimed to study how rhEPO modulates the early *in vivo* effects of RT on colorectal cancer microvasculature. Undoubtedly, modulation of RT effects is mediated by both increased oxygenation and direct effects of rhEPO on normal and tumour microvessels. Moreover, solid tumours are characterised by an important heterogeneity with both well oxygenated and hypoxic or necrotic regions. Therefore, noninvasive functional imaging was used that allows differentiating between different tumour regions.

Expression of the EPO-R was present in all tumours and significantly more pronounced in the hypoxic tumour core. There is at present no clearly defined relationship between tumour hypoxia and expression of the EPO receptor by cancer cells. In head and neck cancer patients, Arcasoy *et al* found a positive correlation between tumour hypoxia and EPO-R expression, whereas others did not observe any correlation in a similar patient cohort (Arcasoy *et al*, 2005; Hoogsteen *et al*, 2005; Winter *et al*, 2005).

The experiment was not intended to study changes in macroscopic tumour growth. We analysed rhEPO-mediated modulation of tumour cell sensitivity to apoptosis. In contrast to the findings of Batra *et al* (2003), we did not observe any difference in expression of apoptotoc or antiapoptotic markers between rhEPO-treated animals and controls. The spatial distribution of apoptotic events did, however, differ in rhEPO-treated animals. In contrast to control animals, Bcl-2 expression in rhEPO-treated animals was significantly different between tumour rim and core, suggesting an increased efficacy of RT in the central region of the tumour by administration of rhEPO.

Dynamic MRI with a macromolecular CA is a validated technique to provide a comprehensive assessment of tumour microvascular physiology (Jackson *et al*, 2005; Preda *et al*, 2006). Pharmacokinetic two-compartment modelling of DCE-MRI data was performed on regions of interest encompassing the tumour vascular rim and the tumour central core. Microvascular plasma flow was significantly decreased by RT in the tumour core (but not in the tumour rim) of both control and rhEPO-treated animals.

In keeping with previous preclinical and clinical findings (Ceelen *et al*, 2006), 5 × 5 Gy of RT decreased endothelial permeability (a surrogate marker for angiogenesis) in the vascular tumour rim of control animals. In rhEPO-treated animals, however, endothelial permeability was unaffected by RT. Similarly, whereas the tumour vascular volume was significantly lower after RT in both the tumour core and rim of control animals, no changes were present in rhEPO-treated animals. This difference in response could be explained both by the previously described direct angiogenic potential of rhEPO counteracting the effect of RT and by the ability of rhEPO to remodel microvessels by an increase in diameter, as confirmed by the microscopy data (cfr infra) (Jaquet *et al*, 2002; Tovari *et al*, 2005).

This modulation of RT effects was accompanied by a significantly lower expression of VEGF in rhEPO-treated animals, a finding previously reported in colorectal xenografts (Tovari *et al*, 2005). As no difference in HIF-1*α* expression was noted, the difference in VEGF expression is likely to result both from better oxygenation of the tumour rim and from direct effects of rhEPO on VEGF expression. Radiotherapy itself has been shown to induce changes in tumour tissue oxygenation. After a single dose of 20 Gy, Ljungkvist *et al* (2006) observed a significant decrease in hypoxic fraction in a murine adenocarcinoma model. We found the increase in oxygenation after 5 × 5 Gy to be spatially heterogenous. Interestingly, in rhEPO-treated animals reoxygenation after RT mainly occurred in the tumour rim, whereas in control animals reoxygenation of the core was more pronounced. Administration of rhEPO resulted in significantly better oxygenation in both tumour regions compared with control animals. This effect is attributable to an increased blood oxygen carrying capacity and has been observed in other preclinical models (Pinel *et al*, 2004).

Erythrocyte flux was measured with the laser Doppler shift method and, in contrast to the modelled plasma flow, provides additional information on rheological properties of the microvasculature. In rhEPO-treated animals, RT did not result in a decrease in LBF, whereas in the central tumour region of control animals, LBF was reduced by RT. This difference in LBF response probably reflects the observed difference in microvessel diameter.

In keeping with others (Hardee *et al*, 2005), we were unable to demonstrate any effect of rhEPO on angiogenesis, as reflected by the similar MVD in both groups. Density of microvessels, however, is only one functional aspect of a tumour microvascular bed. Aspects such as morphology (tortuosity, branching pattern, microvessel diameter), maturation, and endothelial wall permeability represent equally important attributes. We found a significantly larger microvessel diameter in rhEPO-treated rats. Moreover, the MFD was significantly lower in the central region of rhEPO-treated rats, suggesting a lower spatial microvessel complexity (Grizzi *et al*, 2005). These findings confirm the observation of Tovari *et al* (2005) that rhEPO can ‘remodel’ tumour microvessels, although their density seems unaffected. The exact mechanism likely involves the direct action of rhEPO on the endothelium rather than indirect effects mediated through changes in oxygenation, as VEGF expression was significantly lower in rhEPO-treated animals compared with control animals.

In conclusion, the effects of RT on colorectal tumour microvascular physiology are spatially heterogeneous and modulated by administration of rhEPO. Treatment with rhEPO prevented RT-induced changes in microvascular permeability and tumour vascular volume, accompanied by a larger microvessel diameter and altered spatial complexity compared with control animals. It is at present unclear whether this microvascular modulation increases or counteracts the antitumoural efficacy of RT in this model. The present experiment does not allow to draw any conclusion on the effects of rhEPO on radiation response of the tumour cell population as opposed to the response of tumour microvessels. However, as rhEPO resulted in an increased oxygenation of the tumour rim, radiation response of the clonogens is likely to be increased by rhEPO, as described by others (Pinel *et al*, 2004).

Further preclinical experiments will have to elucidate the multiple molecular effects of rhEPO and the interaction with RT on tumour cells, neoplastic endothelium, and normal endothelium.

## References

[bib1] Arcasoy MO, Amin K, Chou SC, Haroon ZA, Varia M, Raleigh JA (2005) Erythropoietin and erythropoietin receptor expression in head and neck cancer: relationship to tumor hypoxia. Clin Cancer Res 11: 20–27 15671524

[bib2] Batra S, Perelman N, Luck LR, Shimada H, Malik P (2003) Pediatric tumor cells express erythropoietin and a functional erythropoietin receptor that promotes angiogenesis and tumor cell survival. Lab Invest 83: 1477–1487 14563949 10.1097/01.lab.0000090156.94795.48

[bib3] Blackwell KL, Kirkpatrick JP, Snyder SA, Broadwater G, Farrell F, Jolliffe L, Brizel DM, Dewhirst MW (2003) Human recombinant erythropoietin significantly improves tumor oxygenation independent of its effects on hemoglobin. Cancer Res 63: 6162–6165 14559797

[bib4] Brurberg KG, Graff BA, Olsen DR, Rofstad EK (2004) Tumor-line specific pO(2) fluctuations in human melanoma xenografts. Int J Radiat Oncol Biol Phys 58: 403–409 14751509 10.1016/j.ijrobp.2003.09.049

[bib5] Ceelen W, El Malt M, Cardon A, Berrevoet F, De Neve W, Pattyn P (2001) Influence of preoperative high-dose radiotherapy on postoperative outcome and colonic anastomotic healing – experimental study in the rat. Dis Colon Rectum 44: 717–721 11357035 10.1007/BF02234573

[bib6] Ceelen W, Smeets P, Backes W, Van Damme N, Boterberg T, Demetter P, Bouckenooghe I, De Visschere M, Peeters M, Pattyn P (2006) Noninvasive monitoring of radiotherapy-induced microvascular changes using dynamic contrast enhanced magnetic resonance imaging (DCE-MRI) in a colorectal tumor model. Int J Radiat Oncol Biol Phys 64: 1188–1196 16457965 10.1016/j.ijrobp.2005.10.026

[bib7] Coppola D, Lu L, Fruehauf JP, Kyshtoobayeva A, Karl RC, Nicosia SV, Yeatman TJ (1998) Analysis of p53, p21(WAF1), and TGF-beta 1 in human ductal adenocarcinoma of the pancreas – TGF-beta 1 protein expression predicts longer survival. Am J Clini Pathol 110: 16–23 10.1093/ajcp/110.1.16 9661918

[bib8] Dey P (2005) Basic principles and applications of fractal geometry in pathology – A review. Anal Quant Cytol Histol 27: 284–290 16447821

[bib9] El-Malt M, Ceelen W, De Meerleer G, Verstraete A, Boterberg T, Van Belle S, de Hemptinne B, De Neve W, Pattyn P (2001) Influence of preoperative combined radiochemotherapy on surgical outcome and colonic anastomotic healing: experimental study in the rat. Int J Radiat Oncol Biol Phys 50: 1073–1078 11429235 10.1016/s0360-3016(01)01600-5

[bib10] Grizzi F, Russo C, Colombo P, Franceschini B, Frezza EE, Cobos E, Chiriva-Internati M (2005) Quantitative evaluation and modeling of two-dimensional neovascular network complexity: the surface fractal dimension. BMC Cancer 5: 14 15701176 10.1186/1471-2407-5-14 PMC549205

[bib11] Hagenaars M, Koelemij R, Ensink NG, van Eendenburg JDH, van Vlierberghe RLP, Eggermont AMM, van de Velde CJH, Fleuren GJ, Kuppen PJK (2000) The development of novel mouse monoclonal antibodies against the CC531 rat colon adenocarcinoma. Clin Exper Metastasis 18: 281–289 11448057 10.1023/a:1011062002851

[bib12] Hardee ME, Kirkpatrick JP, Shan S, Snyder SA, Vujaskovic Z, Rabbani ZN, Dewhirst MW, Blackwell KL (2005) Human recombinant erythropoietin (rEpo) has no effect on tumour growth or angiogenesis. Br J Cancer 93: 1350–1355 16288305 10.1038/sj.bjc.6602846 PMC2361536

[bib13] Harrison L, Blackwell K (2004) Hypoxia and anemia: factors in decreased sensitivity to radiation therapy and chemotherapy? Oncologist 9: 31–40 15591420 10.1634/theoncologist.9-90005-31

[bib14] Henderson E, Sykes J, Drost D, Weinmann HJ, Rutt BK, Lee TY (2000) Simultaneous MRI measurement of blood flow, blood volume, and capillary permeability in mammary tumors using two different contrast agents. J Magn Reson Imaging 12: 991–1003 11105041 10.1002/1522-2586(200012)12:6<991::aid-jmri26>3.0.co;2-1

[bib15] Henke M, Laszig R, Rube C, Schafer U, Haase KD, Schilcher B, Mose S, Beer KGT, Burger U, Dougherty C, Frommhold H (2003) Erythropoietin to treat head and neck cancer patients with anaemia undergoing radiotherapy: randomised, double-blind, placebo-controlled trial. Lancet 362: 1255–1260 14575968 10.1016/S0140-6736(03)14567-9

[bib16] Hoogsteen IJ, Peeters WJM, Marres HAM, Rijken PFJW, van den Hoogen FJA, van der Kogel AJ, Kaanders JHAM (2005) Erythropoietin receptor is not a surrogate marker for tumor hypoxia and does not correlate with survival in head and neck squamous cell carcinomas. Radiother Oncol 76: 213–218 16112214 10.1016/j.radonc.2005.06.030

[bib17] Hylton N (2006) Dynamic contrast-enhanced magnetic resonance imaging as an imaging biomarker. J Clin Oncol 24: 3293–3298 16829653 10.1200/JCO.2006.06.8080

[bib18] Jackson EF, Esparza-Coss E, Bankson A, Coxon A, Patel V, Polverino T, Radinsky R, Starnes C (2005) The effect of AMG 706, a novel tyrosine kinase inhibitor, on vascular permeability and blood flow as assessed by dynamic contrast enhanced magnetic resonance imaging (DCE-MRI) in an *in vivo* preclinical tumor model. J Clin Oncol 23(Suppl): 3134

[bib19] Jaquet K, Krause K, Tawakol-Khodai M, Geidel S, Kuck KH (2002) Erythropoietin and VEGF exhibit equal angiogenic potential. Microvasc Res 64: 326–333 12204656 10.1006/mvre.2002.2426

[bib20] Jelkmann W, Wagner K (2004) Beneficial and ominous aspects of the pleiotropic action of erythropoietin. Ann Hematol 83: 673–686 15322761 10.1007/s00277-004-0911-6

[bib21] Johnson JA, Wilson TA (1966) A model for capillary exchange. Am J Physiol 210: 1299–1303 5923068 10.1152/ajplegacy.1966.210.6.1299

[bib22] Kirkpatrick JP, Hardee ME, Snyder SA, Peltz CM, Zhao YL, Brizel DM, Dewhirst MW, Blackwell KL (2006) The effect of darbepoetin alfa on growth, oxygenation and radioresponsiveness of a breast adenocarcinoma. Radiat Res 165: 192–201 16518899 10.1667/rr3499.1

[bib23] Leyland-Jones B (2003) Breast cancer trial with erythropoietin terminated unexpectedly. Lancet Oncol 4: 459–460 12901958 10.1016/s1470-2045(03)01163-x

[bib24] Littlewood TJ, Bajetta E, Nortier JWR, Vercammen E, Rapoport B, Grp EAS (2001) Effects of epoetin alfa on hematologic parameters and quality of life in cancer patients receiving nonplatinum chemotherapy: results of a randomized, double-blind, placebo-controlled trial. J Clin Oncol 19: 2865–2874 11387359 10.1200/JCO.2001.19.11.2865

[bib25] Ljungkvist ASE, Bussink J, Kaanders JHAM, Wiedenmann NE, Vlasman R, van der Kogel AJ (2006) Dynamics of hypoxia, proliferation and apoptosis after irradiation in a murine tumor model. Radiat Res 165: 326–336 16494521 10.1667/rr3515.1

[bib26] Maiese K, Li FQ, Chong ZZ (2005) New avenues of exploration for erythropoietin. J Am Med Assoc 293: 90–95 10.1001/jama.293.1.90 PMC2254180 15632341

[bib27] Munstedt K, Volzing M, Von Georgi R (2004) Hemoglobin levels during radiation therapy and their influence on local control and survival of patients with endometrial carcinoma. Oncol Rep 11: 711–717 14767527

[bib28] Okano M, Suga H, Masuda S, Nagao M, Narita H, Ikura K, Sasaki R (1993) Characterization of erythropoietin isolated from rat serum – biochemical-comparison of rat and human erythropoietins. Biosci Biotech Biochem 57: 1882–1885 10.1271/bbb.57.1882 7764337

[bib29] Pinel S, Barberi-Heyob M, Cohen-Jonathan E, Merlin JL, Delmas C, Plenat F, Chastagner P (2004) Erythropoietin-induced reduction of hypoxia before and during fractionated irradiation contributes to improvement of radioresponse in human glioma xenografts. Int J Radiat Oncol Biol Phys 59: 250–259 15093922 10.1016/j.ijrobp.2003.12.022

[bib30] Port M, Corot C, Raynal I, Idee JM, Dencausse A, Lancelot E, Meyer D, Bonnemain B, Lautrou J (2001a) Physicochemical and biological evaluation of P792, a rapid-clearance blood-pool agent for magnetic resonance imaging. Invest Radiol 36: 445–454 11500594 10.1097/00004424-200108000-00002

[bib31] Port M, Corot C, Rousseaux O, Raynal I, Devoldere L, Idee JM, Dencausse A, Le Greneur S, Simonot C, Meyer D (2001b) P792: a rapid clearance blood pool agent for magnetic resonance imaging: preliminary results. Magn Reson Mater Phys Biol Med 12: 121–127 10.1007/BF02668093 11390267

[bib32] Pradel C, Siauve N, Bruneteau G, Clement O, de Bazelaire C, Frouin F, Wedge SR, Tessier JL, Robert PH, Frija G, Cuenod CA (2003) Reduced capillary perfusion and permeability in human tumour xenografts treated with the VEGF signalling inhibitor ZD4190: an *in vivo* assessment using dynamic MR imaging and macromolecular contrast media. Magn Reson Imaging 21: 845–851 14599534 10.1016/s0730-725x(03)00186-3

[bib33] Preda A, van Vliet M, Krestin GP, Brasch RC, van Dijke CF (2006) Magnetic resonance macromolecular agents for monitoring tumor microvessels and angiogenesis inhibition. Invest Radiol 41: 325–331 16481916 10.1097/01.rli.0000186565.21375.88

[bib34] Rades D, Tribius S, Yekebas EF, Bahrehmand R, Wildfang I, Kilic E, Muellerleile U, Gross E, Schild SE, Alberti W (2006) Epoetin alfa improves survival after chemoradiation for stage III esophageal cancer: Final results of a prospective observational study. Int J Radiat Oncol Biol Phys 65: 459–465 16584851 10.1016/j.ijrobp.2005.12.019

[bib35] Sabo E, Boltenko A, Sova Y, Stein A, Kleinhaus S, Resnick MB (2001) Microscopic analysis and significance of vascular architectural complexity in renal cell carcinoma. Clin Cancer Res 7: 533–537 11297244

[bib36] St Lawrence KS, Lee TY (1998) An adiabatic approximation to the tissue homogeneity model for water exchange in the brain: I. Theoretical derivation. J Cer Blood Flow Metabol 18: 1365–1377 10.1097/00004647-199812000-00011 9850149

[bib37] Stuben G, Pottgen C, Knuhmann K, Schmidt K, Stuschke M, Thews O, Vaupel P (2003) Erythropoietin restores the anemia-induced reduction in radiosensitivity of experimental human tumors in nude mice. Int J Radiat Oncol Biol Phys 55: 1358–1362 12654448 10.1016/s0360-3016(03)00012-9

[bib38] Thews O, Koenig R, Kelleher DK, Kutzner J, Vaupel P (1998) Enhanced radiosensitivity in experimental tumours following erythropoietin treatment of chemotherapy-induced anaemia. Br J Cancer 78: 752–756 9743294 10.1038/bjc.1998.572 PMC2062974

[bib39] Tovari J, Gilly R, Raso E, Paku S, Bereczky B, Varga N, Vago A, Timar J (2005) Recombinant human erythropoietin alpha targets intratumoral blood vessels, improving chemotherapy in human xenograft models. Cancer Res 65: 7186–7193 16103069 10.1158/0008-5472.CAN-04-2498

[bib40] van Halteren HK, Houterman S, Verheij C, Lemmens V, Coebergh JWW (2004) Anaemia prior to operation is related with poorer long-term survival in patients with operable rectal cancer. Eur J Surg Oncol 30: 628–632 15256236 10.1016/j.ejso.2004.04.014

[bib41] Varlotto J, Stevenson MA (2005) Anemia, tumor hypoxemia, and the cancer patient. Int J Radiat Oncol Biol Phys 63: 25–36 16111569 10.1016/j.ijrobp.2005.04.049

[bib42] Weidner N, Semple JP, Welch WR, Folkman J (1991) Tumor angiogenesis and metastasis – correlation in invasive breast-carcinoma. N Engl J Med 324: 1–8 10.1056/NEJM199101033240101 1701519

[bib43] Winter SC, Shah KA, Campo L, Turley H, Leek R, Corbridge RJ, Cox GJ, Harris AL (2005) Relation of erythropoietin and erythropoietin receptor expression to hypoxia and anemia in head and neck squamous cell carcinoma. Clin Cancer Res 11: 7614–7620 16278379 10.1158/1078-0432.CCR-05-1097

[bib44] Yasuda Y, Fujita Y, Matsuo T, Koinuma S, Hara S, Tazaki A, Onozaki M, Hashimoto M, Musha T, Ogawa K, Fujita H, Nakamura Y, Shiozaki H, Utsumi H (2003) Erythropoietin regulates tumour growth of human malignancies. Carcinogenesis 24: 1021–1029 12807756 10.1093/carcin/bgg060

